# 59. Impact of Pharmacist-Led De-Escalation of MRSA Therapy Using MRSA Nasal PCR

**DOI:** 10.1093/ofid/ofab466.261

**Published:** 2021-12-04

**Authors:** Marc Salvatus, Hemi Jung, Romic Eskandarian, Su Lee

**Affiliations:** 1 West Coast University School of Pharmacy, Sunland, California; 2 Adventist Health Glendale, Glendale, California

## Abstract

**Background:**

Experts suggest that a highly sensitive MRSA nasal PCR can be used to rule out MRSA pneumonia with a negative predictive value greater than 95%. At Adventist Health Glendale (AHGL), the MRSA nasal PCR had a 97% negative predictive value. Pharmacist-led de-escalation of MRSA therapy using MRSA nasal PCR may reduce unnecessary MRSA coverage, patient adverse events, and medication costs. The purpose of this study was to assess the impact on duration of antimicrobial therapy of pharmacist-driven de-escalation of MRSA-targeted antibiotics in pneumonia using MRSA nasal PCR.

**Methods:**

This was a prospective quasi-experimental study (Oct 2018 – Mar 2019 vs. Oct 2019 – Mar 2020) at AHGL, a 515-bed acute care community hospital in Los Angeles, CA, which included adults on MRSA pneumonia agents (either IV vancomycin or IV/PO linezolid). Upon receiving CPOE orders of these MRSA-targeted therapies for pneumonia, the pharmacist ordered a MRSA nasal PCR per protocol for eligible patients, followed up with the results of the MRSA nasal PCR, and recommended to discontinue MRSA therapy if the MRSA nasal PCR was negative. This study received an exemption determination from AHGL IRB.

**Results:**

The total number of patients in the pre-protocol group was 97, and 155 in the post-protocol group. There was a statistically significant decrease in the median duration of MRSA pneumonia agents from the pre-protocol group compared to the post-protocol group (3 days vs. 2 days, P-value = 0.0004). Additionally, there was a statistically significant decrease in the median hospital length of stay from the pre-protocol group compared to the post-protocol group (9 days vs. 7 days, P-value = 0.02).

**Conclusion:**

Implementation of a protocol involving pharmacist-led de-escalation of MRSA-targeted antibiotics for pneumonia utilizing MRSA nasal PCR led to significant decreases in both duration of therapy of MRSA-targeted antibiotics and length of hospital stay.

**Disclosures:**

**All Authors**: No reported disclosures

